# Understanding the underestimation of self-report weight: The roles of narcissism and accountability

**DOI:** 10.1371/journal.pone.0312691

**Published:** 2024-12-02

**Authors:** Menna Price, Laura Douglas, Erica G. Hepper, Laura L. Wilkinson

**Affiliations:** 1 Department of Psychology, Swansea University, Singleton Park, Swansea, United Kingdom; 2 School of Psychology, University of Surrey, Guildford, Surrey, United Kingdom; Australian Catholic University, AUSTRALIA

## Abstract

Self-reported height and weight is widely used to calculate Body Mass Index (BMI) and yet little is known about factors that affect accuracy. This study investigated the motivational characteristics–narcissism and social desirability—that influence the accuracy of self-reported weight and how they interact with accountability (telling participants that their weight will be verified). A two-way between-subjects design was used (accountable vs not accountable) with motivational moderators (narcissism and social desirability). Participants (*N = 80; Mean Age = 34*.*63; 58*.*8% Female*) were randomly allocated to accountable (told that their weight would be verified in a follow-up lab session) or not-accountable (no information given) conditions. In Session 1, participants self-reported motivational (social desirability and narcissism) and anthropometric (height and weight) measures online. In Session 2 (24 hours later), objective measures of height and weight were taken in the lab. There was a significant interaction between condition and maladaptive narcissism level. Being told that weight would be later verified improved accuracy of self-reported weight, but only for those low in maladaptive narcissism. Accountability improves the accuracy of self-report weight data, but not for individuals high in narcissism. Though based on a modest sample, these findings suggest that the under-estimation of self- report weight serves a self-protective function and maladaptive narcissism may be a useful covariate to include in research using self-report weight estimates.

## Introduction

Self-report methods for collecting anthropometric data are in common use in weight-related research [1]. It is a practical and cost-effective way to obtain large datasets [2] and self-reported height and weight correlate strongly with objective measures [3]. However, weight is generally under-reported and height over-reported [4,5] resulting in biased estimates of weight status [6]. Such evidence calls into question the reliability and validity of self-report data, potentially undermining the interpretation of associated results. However, one aspect that remains relatively unexplored is the standard deviation of discrepancies between self-reported and measured data, which suggest variability between individuals. Understanding the individual differences that underlie weight under-reporting is the first step towards improving the accuracy of self-report data and appropriately interpreting results that use this approach.

Suggestions that weight under-estimation may be the result of measurement error, ignorance or guesswork [7] are undermined by the fact that self-reported weight becomes more accurate when individuals are aware that their estimates will be verified [8,9]. The fact that being held “accountable” for self-report data improves accuracy suggests that weight under-estimation may serve a motivational role and this may be a promising target for improving accuracy. Polivy, Herman, Trottier and Sidhu [10] reviewed relevant literature and proposed that the two most likely *motivational* reasons to under-report weight are 1) impression management (i.e., presenting to others in a socially desirable way) and 2) self-enhancement/protection (i.e., holding unrealistically positive beliefs about the self to preserve self-esteem). Although a number of studies have investigated the relationship between social desirability and self-reported weight accuracy [11], few have examined the role of self-enhancement/protection [12] or their interaction with accountability. Indeed, DeAndrea et al. [13] manipulated accountability in a student sample and found that although accountability significantly reduced weight discrepancies, a proportion of the sample (23%) still under-estimated their weight. The authors suggest that this is unlikely to be related to impression management, as it is not socially desirable to give inaccurate information. Instead, it may be due to avoidance of threats to the self-esteem (self-enhancement/protection), but this hypothesis is yet to be tested. Therefore, the present study aims to explore these three key concepts and how they might theoretically relate to each other (explored below in turn).

Anthropometric measurements, like all self-report measures, are susceptible to response bias, particularly social desirability bias. Socially desirable responding can be defined as the tendency to give self-descriptions that convey a positive rather than an accurate view of oneself to others–impression management [14]. Individuals are inclined to present more favourable images of themselves to conform or gain social approval [15]. In Western cultures, body image is considered a core aspect of both physical and mental well-being [16], thus body weight and health status can be considered an area in which individuals strive to meet socially desirable norms. In accordance with this notion, social desirability bias has been shown to influence self-report accuracy in several studies investigating health behaviours related to weight, including dietary intake [17] and physical exercise [18]. Indeed, women who score high in the tendency to respond in a socially desirable manner are most likely to under-report their weight [19]. Moreover, the degree of bias is negatively associated with the discrepancy between measured body weight and perceived socially ideal weight [20]. Therefore, those with strong social desirability tendencies are most likely to under-report their weight.

However, in all of the above studies participants were unaware that their weight would be verified. It is currently unknown whether individuals high in social desirability might report more honest and accurate estimates when they know they will be held accountable later. Given that honesty and accuracy are also socially desirable traits, they might even be more accurate than low-social desirability individuals under accountable conditions. Of note, previous research has made use of a general measure of social desirability [21], but this does not specifically tap into impression management. The Balanced Inventory of Desirable Responding (BIDR; [22] on the other hand, distinguishes between impression management and self-deceptive enhancement. Whereas impression management is a direct measure of the tendency to intentionally distort responses in order to present a positive social image, self-deceptive enhancement measures overconfidence in one’s judgements and therefore favourably biased responses are presumed to be honest. It is the impression management subscale of the BIDR (and not the self-deceptive enhancement subscale) that is sensitive to self-reported information provision via online platforms [23] and so this subscale was of particular interest in the current study.

Self-enhancement and self-protection reflect the motives to boost and protect positive views of the self, respectively [24,25]. Although the two motives can manifest in distinct patterns of thought, feeling, and behaviour, they operate in conjunction and are often indistinguishable to an individual and to researchers. In the context of weight under-estimation, Polivy et al. [26] argued that both self-enhancing and self-protecting patterns are apparent and that the motives may be impossible to disentangle. However, both motives are strongly predicted by the personality trait of narcissism [27]. Thus, when researchers seek to capture individual differences in self-enhancing/protective tendencies narcissism is a key indicator variable.

Narcissism is a multifaceted nonclinical personality trait that comprises grandiose self-views, a desire for admiration and power, and lack of communal orientation and empathy [28,29]. The trait shares characteristics with Narcissistic Personality Disorder but is empirically distinct and varies continuously and normally in the general population [30,31]. Individuals high in nonclinical narcissism have a tendency to build a highly positive and grandiose sense of self, termed as building the Taj Mahal of selves [32]. Although self-enhancement and self-protection are universal motives, high-narcissists rely on such strategies to a greater or even excessive degree to maintain self-esteem and their grandiose identity [33,34]. Consistent evidence documents high-narcissists’ tendency to over-report their attributes and abilities [35]. Particularly relevant for our current study, is the finding that narcissistic individuals are likely to base their self-worth on looks [36] and have been shown to overestimate their own attractiveness [37]. Given that BMI is considered an indicator of attractiveness [38], it is reasonable to suggest that those high in narcissism may be more susceptible to inaccurately report their height and weight in order to confirm their grandiose self-view. A further consideration is the component dimensions within narcissism. So far, research on self-reported attractiveness has focused on overall narcissism level. However, narcissism comprises both relatively socially adaptive (e.g., leadership, self-sufficiency) as well as socially maladaptive dimensions (e.g., exploitativeness, entitlement, exhibitionism) [39,40]. This distinction has been theorised to reflect a relative focus on assertive self-enhancement vs. antagonistic self-protection [41] and so disentangling the roles of adaptive and maladaptive narcissism dimensions may shed light on the relative prominence of enhancing vs. protective biases in weight under-estimation.

Overall, accountability curtails self-enhancement and reduces weight under-estimation [42]. However, individuals high in narcissism are less responsive to social norms for modesty [43] and may continue to self-enhance regardless of the cost to social relationships [44]. Consistent with this prediction, Collins and Stukas [45] found that holding participants accountable (by requiring them to justify self-ratings of attributes to an expert) led to more modest self-ratings among low-narcissistic individuals but actually *inflated* self-ratings among high-narcissists, especially when the domains were personally important to them. Again, dimensions within narcissism may be informative. Given that adaptive narcissism relates to leadership proclivities and likeability [46] whereas maladaptive narcissism relates to low empathy and peer-reported aggression [47,48], it seems likely that maladaptive narcissism would drive the tendency to retain biased self-reports even when accountable. Therefore, it is critical that not only accountability and narcissism are studied when investigating factors that may influence the accuracy of self-reported data, but that their interaction is taken into account too.

The aim of the current study was to contribute to the understanding of motivational factors underlying the accuracy of weight self-reports. Specifically, we investigated how the traits of impression management and narcissistic self-enhancement/protection predict self-reported weight accuracy, and whether any such associations are visible when participants do and do not know that their self-reports will be verified (i.e., varying conditions of accountability). It was predicted that those with higher impression management and narcissism scores will be more likely to under-estimate their weight and over-estimate their height when self-reporting. Further, this was expected to interact with accountability, such that those high in impression management would only under-estimate their weight when they do not know that their weight will be later verified (were not told that they would be weighed objectively the following day). Conversely, those high in narcissism, particularly maladaptive narcissism, would under-estimate their weight regardless of whether their weight would be objectively measured later.

## Method

### Participants

Eighty volunteers were recruited from students in a UK University and local community members who participated without reward. The sample comprised 33 men and 47 women aged between 18–82 years (see Table 1 for sample characteristics). Sample size was based on previous research that has investigated differences in weight estimation in accountable versus non-accountable groups (DeAndrea et al, 2012; N = 78). Participants with a historical or current diagnosis of an Eating Disorder were excluded from taking part in the study. The study recruitment period was between 15^th^ July– 15^th^ August, 2016. Data collection was carried out by a single researcher and ceased once 80 participants had completed both stages of the study.

**Table 1:** Sample characteristics.

	Whole Sample N = 80	Accountable Group N = 39	Non-accountable Group N = 41
Age (Years: Mean (SD))	34.63 (16.32)	34.87 (16.7)	34.39 (16.16)
BMI (Mean (SD))	26.19 (4.78)	26.50 (5.24)	25.90 (4.34)
% Female	58.8%	53.8%	63.4%

Note: Groups did not differ significantly by age, BMI or gender (p>.05). Accountable Group = told they would be weighed objectively later; Non-accountable Group = Not told they would be weighed objectively later.

Ethics Statement: The study was granted ethical approval by the University’s Department of Psychology Research Ethics Committee. Written informed consent was obtained from all participants. This is in accordance with the 1975 Declaration of Helsinki (as revised in 2013).

### Procedure

Participants were invited to take part in a study on “Factors influencing how we value food”. The study comprised two sessions, 24 hours apart. In Session 1, participants completed a questionnaire (online or on paper as preferred). Participants were randomly allocated to one of two conditions. In the *accountable* condition, the information sheet stated that their height and weight would be measured in Session 2. In the *non-accountable* condition, this information was omitted. After providing consent via an online form, participants were then asked to self-report their demographic variables, height and weight, and then completed the personality questionnaires.

For Session 2, participants were invited into the laboratory and height and weight were measured and recorded by the experimenter, followed by several computer tasks that supported the cover story but were unrelated to the purpose of this investigation. Afterward, participants were asked to complete two demand awareness questions, asking what they believed the aim of the study was and whether they believed they had been told their height and weight would be measured. Subsequently all participants were thanked and debriefed.

### Measures

#### Balanced Inventory of Desirable Responding Short Form (BIDR-16)

The BIDR measures two factors of socially desirable responding: impression management (IM) and self-deceptive enhancement (SDE). It is a robust measure with adequate internal consistency and test-retest reliability [49,50]. Each subscale comprises 8 items (1 = strongly disagree, 8 = strongly agree) which are summed, with higher scores indicative of more socially desirable responding. Though internal consistency is relatively low (not always exceeding a Cronbach’s alpha of .70), it is a well-used measure in the literature and these alpha levels are consistent with comparable measures (e.g. BIDR-40). The BIDR entails a broad range of self-enhancement and Impression Management instantiations and this is reflected in the alpha values. Both subscales were distributed, but the analysis focused on IM as it captures the distortion of responses in order to present a positive social image, which was the primary motivation of interest.

#### Narcissistic Personality Inventory (NPI)

The NPI [51] is the most widely-used measure of nonclinical narcissism [52]. In each of 40 forced-choice items, participants are shown two statements (one narcissistic, one non-narcissistic) and asked to select the statement that best describes them. The number of narcissistic choices is summed, with higher scores indicative of higher narcissism. Two factor-based subscales were also derived for analysis: *adaptive narcissism* comprises Raskin and Hall’s Authority and Self-Sufficiency items, and *maladaptive narcissism* comprises Exploitativeness, Entitlement, and Exhibitionism items [53]. NPI adaptive and maladaptive sub-scales are not always high (sometimes with a Cronbach alpha value of just below .70), however, this is typical in the literature and they are well-used sub-scales [48]. The NPI shows high test-retest reliability (*r* = .80; [54] and its convergent and predictive validity are well established [55].

#### Anthropometric measurements

During the first session of the study participants were asked to self-report their height and weight (in cm and kg). In Session 2, height and weight was measured by the researcher. Subjects wore no shoes and light clothing during measurements. Height was measured to the nearest 0.5 cm using a free-standing stadiometer. Weight was measured to the nearest 0.1 kg using standardized digital weighing scales.

#### BMI and gender

Gender and Body Mass Index (BMI) were recorded as they are important baseline characteristics when examining self-report height and weight. Higher BMI has been found to be predictive of greater weight underestimation [56], with the heaviest women under-reporting their weight and the lightest over-reporting [57]. Whereas women view weight as a principal determinant of their body image [58], tall height is of central importance to men’s body image [59]. Consistent with this notion, research has illustrated that women underestimate their weight by larger discrepancies than men [60,61], and men overestimate their height to a greater extent than women [62,63].

## Results

### Preliminary analyses: Self-reported and measured height, weight and BMI

Self-reported and measured height, weight and BMI were normally distributed (*Z*skew < 2). Related *t*-tests showed that self-reported weight and BMI (but not height) were significantly under-estimated; moreover, self-reported and measured height, weight and BMI were significantly, positively correlated (Table 2). Weight discrepancy (WD) was calculated as the difference between self-reported and measured weight in kg, such that a higher value indicates greater under-reporting of weight. Gender (coded Male = 1, Female = 2) (*r* = .01; *p* = .91) did not correlate with WD in this sample. However, measured BMI was positively and significantly related to WD (*r* = .40; *p* < .0001). Therefore, BMI was used as a covariate in all subsequent analyses. All analyses were conducted using SPSS software (version 22.0).

**Table 2:** Difference and correlation test outcomes for self-report and measured anthropometric data.

	Physical Characteristics (Mean ± SD)		Related t-test			Correlation	
** **	Self-Report	Objective Measure	t	p	*d*	r	p
**Height (cm)**	170.18 (±10.54)	170.02 (±10.36)	0.57	0.57	.02	.97	<0.001
**Weight (kg)**	73.71 (±13.12)	75.50 (±13.83)	-4.84	<0.001	.13	.97	<0.001
**BMI (kg/m** ^**2**^ **)**	25.50 (±4.32)	26.19 (±4.78)	-4.46	<0.001	.14	.96	<0.001

### Accountability, impression management, narcissism and weight discrepancy

**Fig 1:**
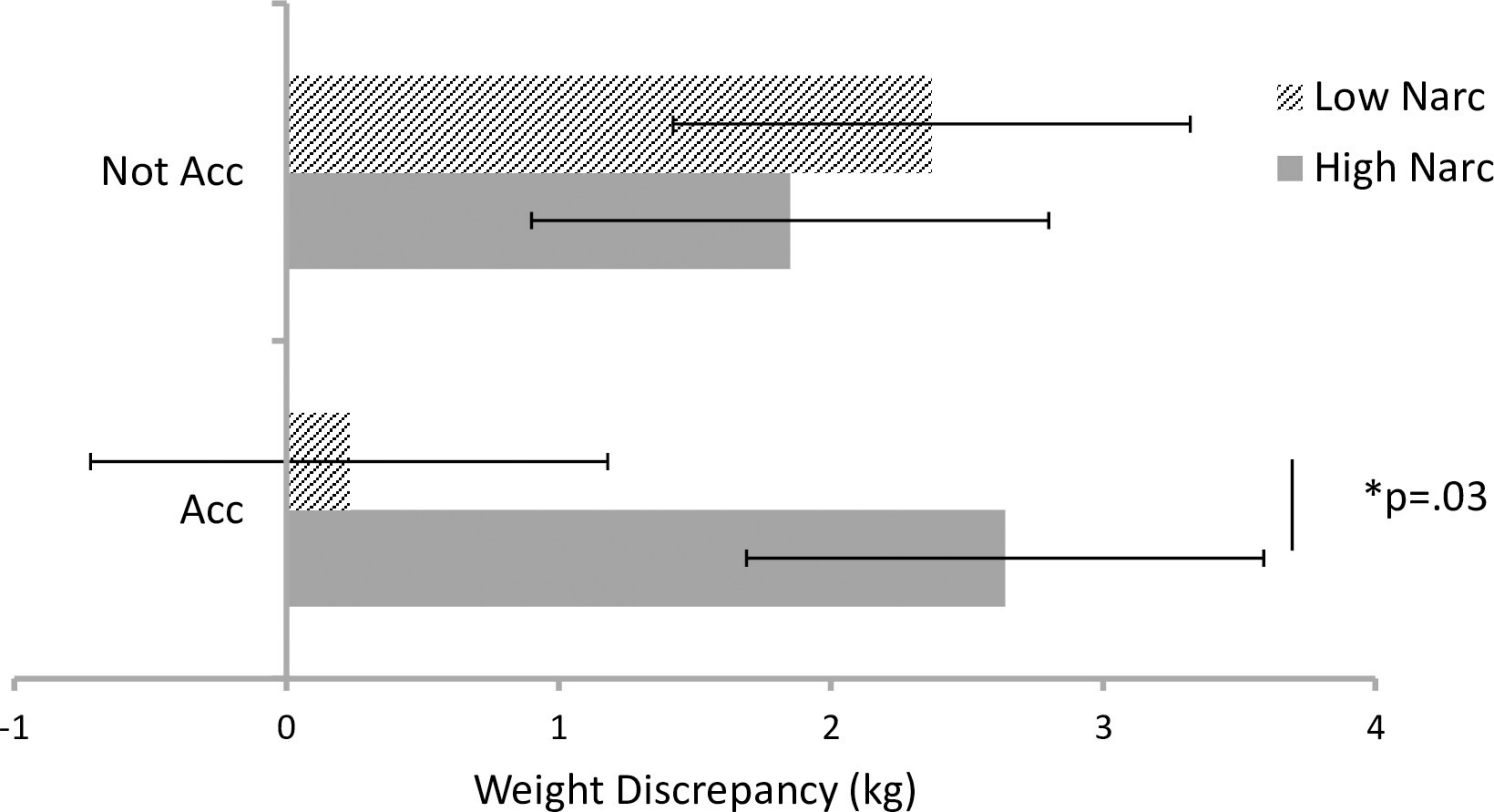
Mean (SE) Weight Discrepancy in the Accountable (Acc) versus Not Accountable (Not Acc) Groups at high and low levels (+ 1 SD) of Maladaptive Narcissism (Narc).

Moderation analyses (PROCESS Version 3.0, Model 1; [64] were used to examine the roles of impression management (IM) and narcissism (maladaptive and adaptive subscales) in the relationship between accountability condition and WD. All analyses controlled for measured BMI, and were mean centred prior to analysis. Moreover, each narcissism model controlled for the other narcissism subscale to identify their unique contributions. Accountability condition, impression management, and narcissism (adaptive and maladaptive) did not individually predict WD (see Table 3); however, there was a significant interaction between accountability and maladaptive narcissism. Simple slopes analysis at 1*SD* above and below the mean on maladaptive narcissism revealed that accountability (being told one’s weight would be objectively verified) reduced WD for those low in maladaptive narcissism (*B* = .40, *t* = 2.24, *p* = .03, CI = 0.23, 4.05), but not for those high in maladaptive narcissism (*B* = -.10, *t* = -.83, *p* = .41, CI = -2.69, 1.11) (see Fig 1). The model remained unchanged if adaptive narcissism was excluded from the model. Adaptive narcissism and impression management did not moderate the effect of accountability (Table 3).

**Table 3:** PROCESS Model 1 moderation output for predicting weight discrepancy.

Subscale	Predictor	B	SE	t	p	(LLCI	ULCI)
IM NPI Malad	Accountability	.64	.71	.90	.372	(-.77	2.04)
	IM	-.01	.06	-.18	.858	(-.13	.11)
	Acc x IM	-.01	.12	-.11	.912	(-.26	.23)
	BMI	.28	.07	3.84	.001***	(.14	.43)
	Accountability	.68	.67	1.01	.314	(-.66	2.01)
	NPI Malad	.15	.14	1.07	.288	(-.13	.43)
	Acc x NPI Malad	-.49	.23	-2.15	.035*	(-.94	-.04)
	BMI	.31	.17	4.27	.001***	(.16	.45)
	NPI Adap	-.11	.17	-.66	.51	(-.44	.22)
NPI Adap	Accountability	.67	.69	.98	.332	(-.70	2.05)
	NPI Adap	-.09	.17	-.58	.563	(-.44	.24)
	Acc x NPI Adap	-.15	.28	-.53	.601	(-.69	.40)
	BMI	.30	.07	3.97	.001***	(.15	.44)
	NPI Malad	.18	.14	1.24	.22	(-.11	.47)

Note: Coeff (Unstandardised coefficient); se (Standard Error); LLCI (Lower Level Confidence Interval); ULCI (Upper Level Confidence Interval); IM (Impression Management); NPI Malad (Narcissistic Personality Inventory Maladaptive Narcissism); NPI Adap (Narcissistic Personality Inventory Adaptive Narcissism); Accountability (Accountable coded as 1 and not accountable coded as 2); BMI (Body Mass Index (measured).

* p < .05

*** p < .001.

### Supplementary analysis

The number of participants who wrongly identified their condition during debrief was recorded. Of the 39 participants in the accountable group, four participants (10.2%) reported that they were not aware that their weight and height would be measured. Of the 41 participants in the non-accountable group, 12 participants (29.3%) incorrectly reported that they *had* been told they would be measured. Therefore, the WD models were conducted again controlling for correct vs. incorrect recall, but this did not alter the results.

## Discussion

The current study examined individual differences in weight underestimation, seeking to better understand the motivational drivers of this behaviour. The main aim of the current study was to investigate the association between motivational traits (impression management and narcissistic self-enhancement) and self-reported weight accuracy, and specifically the interaction of these traits with accountability. Results showed that maladaptive narcissism (i.e., the tendency to self-protect in exploitative, entitled and exhibitionistic ways) moderated the effect of accountability condition: being told that weight would be later verified improved the accuracy of self-reported weight, but only for individuals who were low in maladaptive narcissism.

Consistent with previous research, self-reported height, weight and BMI were strongly and positively correlated with observed measures across the sample [65]. The current findings also confirmed previous findings [66] that self-reported weight was significantly lower than observed weight across the sample. However, no differences were found between self-reported and observed height. Although general trends in the literature show over-reporting of height, this is variable between studies and may depend on other as yet unidentified demographic factors [67].

Our results showed that accountability only affected those who were low in narcissism. Narcissism reflects a strong reliance on self-serving biases to boost and maintain grandiose self-views [68], and the present findings add to extensive prior evidence that high-narcissists inflate self-reports of their attributes [69]. It is noteworthy that in the present non-accountable condition, participants underestimated their weight regardless of their narcissism levels, reflecting the commonplace and universal nature of self-enhancing and self-protective biases [70]. However, whereas low-narcissists responded to accountability by increasing accuracy of their reports, those high in maladaptive narcissism preserved their biased reports regardless of consequences.

The presence of this pattern for maladaptive (but not adaptive) narcissism extends understanding of the specific self-motives underlying weight underestimation (cf. [71]. Maladaptive narcissism dimensions are associated with low communal orientation and defensive, antagonistic self-protection [72–74]. Accordingly, the present findings imply that weight underestimation may reflect defensive self-protection (i.e., avoiding negative weight-related perceptions) more than self-enhancement (i.e., seeking perceptions of oneself as more desirable). Alternatively, the findings might reflect maladaptive narcissists’ lack of concern for others and consequent lack of regard for modesty norms. Given that impression management did not show a parallel pattern, however, this interpretation seems less plausible. Future research could test these possibilities more directly using measures of different self-enhancing/protective proclivities or manipulating the framing of weight-related information. The present findings also support evidence that narcissistic individuals are unlikely to respond to calls for accuracy over self-enhancement when it comes to their physical appearance as they are more likely to base their self-worth on their looks [75]. Given current indications that narcissistic tendencies are on the rise in younger generations [76], this is a vital characteristic to take into account in future health research that makes use of self-report data. Including maladaptive narcissism scores as a covariate would allow researchers to adjust for anticipated weight under-estimations in this group and would facilitate greater accuracy.

Impression management did not predict WD or moderate the impact of accountability. This suggests that the motivation to underestimate weight serves a self-protective function rather than an impression management function. Such an interpretation is consistent with Polivy et al.’s [77] review conclusion that weight discrepancies in women who were dieting and restrained-eaters were the consequence of self-protection mechanisms rather than impression management. As this had only been previously found in women who were engaging in dietary restraint (who are more likely to under-estimate their weight), the current findings add to the literature by showing that this extends to a population that also includes men and non-dieting women. Furthermore, the current sample had a wide age range (18–82 years) allowing for the results to be generalisable across adulthood. Although our sample is broader than those used previously in related research, we note that we did not record ethnicity and therefore we cannot assume that the findings generalise across ethnicity and culture. For example, we cannot assume that weight underestimation is consistent across ethnic groups and cultures given the Westernised ‘Thin-Ideal’. To this end, future research should include cross cultural samples and consideration of body image dissatisfaction and related self-esteem within those samples. We may well expect to find totally different patterns in societies that don’t endorse the thin-ideal, given that we interpret under-estimation as self-enhancement and so in a different culture, different misreports of weight or appearance might instead be self-enhancing. Moreover, individuals in collectivistic cultures tend to rely on different types of self-enhancement strategy, prioritising chronic flattering private beliefs over public self-presentation [78] and so we might also see less difference between accountability conditions in some cultures.

Contrary to previous research, we did not find a significant main effect of accountability, where making people accountable for their self-reported weight improved accuracy [79,80]. One possibility for the inconsistency is that their sample consisted of low narcissistic individuals, but this was not measured. Examination of the mean differences in WD scores between accountable versus non-accountable groups in Black et al [81] shows that they are comparable to those found in the current study (0.48KG and 0.49KG respectively).

## Conclusions

Weight is consistently under-reported using self-report measures. Although it correlates strongly with observed weight, the implications for research requiring specific weight and BMI estimates, such as in weight interventions, are critical. The findings here suggest that accountability can improve self-report estimates (as suggested by previous research) but demonstrate for the first time that this is only the case for individuals who are low in maladaptive facets of narcissism. For individuals higher in narcissism, the motive to protect self-esteem via maintaining biased weight perceptions seems to dominate and override accuracy even when accountable to objective measurement. The recent increase in narcissistic tendencies in society [82] highlights the need to examine this trend longitudinally and take this trait into consideration when using self-report data in health research in the future.
